# Supplementary material for: Complexome profiling of the Chlamydomonas psb28 mutant reveals TEF5 as an early PSII assembly factor
Source: Plant Cell. 2025 Mar 17;37(6):koaf055. doi: 10.1093/plcell/koaf055 (PMC12164590; doi:10.1093/plcell/koaf055)
Supplement: koaf055_Supplementary_Data [file koaf055_supplementary_data.zip › psb28_interactive.zip!/plots/intensity/qualityPlots/peptides/RSEP2_Cre03.g144164.t1.1.html]

### Cre03.g144164.t1.1

Trivial name: RSEP2  
SeqCoverage [median]: 19.80  
Mass: 51.60 kDa  
  
 

| Sequence | MissedCleavage | Mass [Da] | wtIntensity | muIntensity | Score |
| --- | --- | --- | --- | --- | --- |
| ELVVEVERPSASAASASGSGSQPGGAEVLQLR | 1 | 3137.60 | 0.00 | 1803.08 | 61.50 |
| LGDVVVPAAPSQVSTSVSAIR | 0 | 2052.12 | 0.00 | 2693.70 | 47.67 |
| MGVQLTSNTYIK | 0 | 1353.70 | 0.00 | 3052.73 | 133.23 |
| SGDIILR | 0 | 772.44 | 428.79 | 3680.52 | 94.41 |
| VTPDAGSDGGGR | 0 | 1087.49 | 1074.16 | 5188.03 | 90.11 |
| VVVPDTPAGAMSAAAR | 0 | 1511.78 | 1401.02 | 2887.45 | 85.85 |
|  |  |  | 2904.0 | 19305.5 |  |
